# Discovery of Novel *APOC3* Isoforms in Hepatic and Intestinal Cell Models Using Long-Read RNA Sequencing

**DOI:** 10.3390/genes16040412

**Published:** 2025-03-31

**Authors:** Kara Farstad-O’Halloran, Anuradha Sooda, Tooba Iqbal, Steve Wilton, May T. Aung-Htut

**Affiliations:** 1 Personalised Medicine Centre, Health Futures Institute, Murdoch University, Murdoch, Perth, WA 6150, Australiaa.sooda@murdoch.edu.au (A.S.); s.wilton@murdoch.edu.au (S.W.); 2 Perron Institute for Neurological and Translational Science, The University of Western Australia, Nedlands, Perth, WA 6009, Australia

**Keywords:** apolipoprotein C-III (*APOC3*), triglyceride metabolism, cardiovascular disease, novel transcript isoforms, long-read RNA sequencing

## Abstract

Background: Apolipoprotein C-III (*APOC3*) plays a crucial role in triglyceride metabolism and is closely associated with cardiovascular disease risk. Elevated APOC3 levels contribute to higher plasma triglycerides and increased risk of atherosclerosis, making *APOC3* expression an attractive and logical therapeutic target. Methods: While studying various *APOC3* transcript isoforms expressed in hepatoma cell lines (HepG2, Huh7) and healthy liver tissue using publicly available long-read RNA sequencing, we found three novel *APOC3* isoforms. These isoforms were validated through RT-PCR and Sanger sequencing. Results: All three novel isoforms are splicing variants of the MANE transcript, *APOC3-201*. Isoforms 1 and 2 exhibit splicing patterns similar to *APOC3-201* from exons 2–4; however, isoform 1 shares its exon 1 splicing pattern with *APOC3-203*, while isoform 2 features an extended exon 1 that includes exon 1a, the adjacent intronic region, and exon 1b. The third isoform closely resembles *APOC3-201*, but lacks exon 2, which contains the translation start codon. Remarkably, similar *APOC3* splicing patterns and transcript variants were observed in Caco-2 cells, a model of the small intestine, indicating that these isoforms are not liver-specific. Conclusions: This study identifies three novel *APOC3* isoforms and highlights their expression in both hepatic and intestinal cell models. Further studies are needed to elucidate the functional roles of these novel isoforms and their contribution to the regulation of *APOC3* gene expression.

## 1. Introduction

Cardiovascular disease (CVD) remains the leading cause of morbidity and mortality worldwide. A well-established association exists between elevated triglyceride levels and an increased risk of CVD [1,2]. Apolipoprotein C-III (APOC3), a critical component of triglyceride-rich lipoproteins (TRLs), plays a central role in regulating lipid metabolism by inhibiting the uptake and clearance of circulating TRLs [3]. This leads to hypertriglyceridemia [4], a well-established risk factor for atherosclerosis and ischemic heart disease. Genetic studies have shown that loss-of-function mutations in the *APOC3* gene are associated with significantly lower triglyceride levels, reduced remnant cholesterol, and less coronary artery calcification, a marker of atherosclerosis [5,6,7]. Additionally, transgenic and knockout mouse models have reinforced these findings, demonstrating that *APOC3* overexpression induces hypertriglyceridemia, whereas its absence promotes efficient lipid clearance and a favourable cardiovascular profile [8,9]. In addition to its role in triglyceride metabolism, *APOC3* has been implicated in type 2 diabetes mellitus (T2DM) pathophysiology through mechanisms such as promoting pancreatic β-cell apoptosis, dysregulation of glucose metabolism, and influencing insulin resistance [10,11,12,13]. Its role in inflammatory processes, including monocyte adhesion and endothelial dysfunction, further emphasizes its significance in the pathogenesis of cardiometabolic diseases [14]. Given these multifaceted roles, targeting *APOC3* presents a promising therapeutic target for lowering cardiovascular risk.

To date, four protein-coding transcripts of *APOC3* have been reported in the Ensembl genome database (release 113) [15]. The primary transcript, *APOC3-201* (ENST00000227667.8), includes all four exons and encodes a 99 amino acid (aa) protein. Another full-length transcript, *APOC3-202* (ENST00000375345.3), closely resembles *APOC3-201* but contains a longer exon 2 sequence, resulting in a 117 aa protein. Two truncated isoforms, *APOC3-203* and *APOC3-205*, have also been described. *APOC3-203* (ENST00000433777.5) utilises an alternate splice donor site in exon 3, leading to the exclusion of exon 4 from the mature transcript. This is predicted to produce a truncated 55 aa protein lacking the C-terminal domain, though experimental evidence to confirm the presence of this peptide is currently lacking. Notably, *APOC3-203* also uses an alternative upstream exon 1 (exon 1a) sequence distinct from the exon 1 (exon 1b) employed by the full-length transcripts. Finally, *APOC3-205* (ENST00000630701.1) contains exons 2 to 4 and is reported to produce a functional 117 aa protein. We detected a novel isoform missing exon 2 in hepatoma cell lines (HepG2 and Huh7) when *APOC3* transcripts were amplified using primers targeting exon 1a/b (forward) and 3 (reverse) and hence further investigated the presence of additional isoforms.

The advancement of high-throughput sequencing technologies and the growing adoption of long-read sequencing platforms have led to a rapid expansion of transcriptomic data. This has provided an opportunity to evaluate full-length transcripts and more accurately identify alternative splicing events, offering insights into previously underexplored areas of gene regulation [16,17]. Such findings are crucial for understanding the regulatory mechanisms governing *APOC3* expression and for identifying additional isoforms that could be involved in lipid metabolism.

In this study, we utilised publicly available long-read RNA-seq data from commonly used hepatoma cell lines (HepG2 and Huh7), liver tissue, and Caco-2 cells, a model of the small intestine, to investigate the isoform diversity of *APOC3*. Through the analysis of these datasets, we identified novel isoforms of *APOC3* and gained valuable insights into its splicing patterns. While lipid metabolism is not our primary area of expertise, our findings provide a foundation for further exploration into the regulation of lipid metabolism and *APOC3* isoforms.

## 2. Materials and Methods

### 2.1. RNA Sequencing Data

The raw RNA sequencing data from Oxford Nanopore used in this study were obtained from the NCBI Sequence Read Archive (SRA) under the following accession numbers: PRJNA765908 (HepG2) [18], PRJNA893571 (Huh7) [19], PRJEB81685 (Liver) [20], and PRJNA850621 (Caco-2) [21] (Supplementary Table S1). Data acquisition was performed using the prefetch tool from the SRA Toolkit (v2.11.3), and raw sequencing reads in FASTQ format were extracted using fastq-dump (https://github.com/ncbi/sra-tools, accessed on 27 October 2023). The resulting FASTQ files were then processed and analysed using computational pipelines optimised for long-read RNA sequencing data.

### 2.2. Bioinformatics Analyses

To identify isoforms of the *APOC3* gene, we applied the Full-Length Analysis of Mutations and Splicing in long-read RNA-seq data (FLAMES) pipeline (https://github.com/LuyiTian/FLAMES, accessed on 16 December 2024) with the default parameters, adjusting “strand_specific” to 1 for direct RNA sequencing and 0 for cDNA sequencing. Additionally, we filtered transcripts to include only those supported by at least 10 reads to minimise technical noise and sequencing errors.

Isoform classification and splicing analysis were performed using SQANTI3 (v5.2) (https://github.com/ConesaLab/SQANTI3, accessed on 16 December 2024) [22]. Gencode human hg38.v47 was used as the gene reference annotation. For the reference files of CAGE Peak data, and polyA motif list, we used the files provided in SQANTI3.

The bioinformatics workflow is summarised in Supplementary Figure S1.

### 2.3. Secondary Structure Prediction

To predict the secondary structures of the 5′ untranslated region (5′UTR) of Novel-2, we used the Vienna RNAfold web server (http://rna.tbi.univie.ac.at, accessed on 25 February 2025) [23]. The analysed region included exon 1a (116,829,706–116,829,886), intron 1 (116,829,887–116,829,906), exon 1b (116,829,907–116,829,940), and exon 2 (116,830,570–116,830,582), spanning a total of 248 bp. For comparison, we included the 5′UTRs of *APOC3-201* (exon 1b: 116,829,907–116,829,940; exon 2: 116,830,570–116,830,582 (exon 2); 47 bp) and *APOC3-203* (exon 1a: 116,829,706–116,829,886; exon 2: 116,830,570–116,830,582; 194 bp). The sequences were input into the RNAfold web interface, and the resulting minimum free energy (MFE) structures were analysed to assess structural stability and potential functional elements.

### 2.4. Phylogenetic Trees

Multiple sequence alignment and phylogenetic tree construction were performed using the MAFFT online server (https://mafft.cbrc.jp/alignment/server/index.html, accessed on 28 January 2025). Phylogenetic trees were generated using the Neighbour–Joining method with the Jukes–Cantor nucleotide substitution model and a bootstrap value of 1000.

### 2.5. Cell Lines and Culture

HepG2 (Cat. #85011430) and Huh-7 (Cat. #JCRB0403) cell lines were purchased from CellBank Australia (Sydney, Australia) and originally supplied by the European Collection of Cell Cultures (Salisbury, UK). The cells were cultured in Dulbecco’s Modified Eagle Medium (Gibco, Thermo Fisher Scientific, Melbourne, Australia; Cat. #11995-065) supplemented with 10% fetal bovine serum (Fisher Biotec, Perth, Australia; Cat. # FBS-AU-015). Cultures were maintained in a humidified incubator at 37 °C with 5% CO_2_ (*v*/*v*).

### 2.6. RNA Extraction

Total RNA was extracted using the MagMAX™ Nucleic Acid Isolation Kit (Thermo Fisher Scientific, Waltham, MA, USA) following the manufacturer’s protocol. RNA concentration and purity were measured with an Implen NanoPhotometer^®^ (Implen NanoPhotometer^®^, Westlake Village, CA, USA).

### 2.7. RT-PCR and Gel Electrophoresis

Reverse-transcription polymerase chain reaction (RT-PCR) was performed using the SuperScript^®^ III One-Step RT-PCR System (Life Technologies, Melbourne, Australia) with 50 ng of RNA as the template. Primer sequences are detailed in Supplementary Table S2. RT-PCR products were separated on a 3% agarose gel in 1× TAE buffer, stained with 1× RedSafe™ (iNtRON Biotechnology, Burlington, MA, USA), and imaged using a Fusion-FX Gel Documentation System (Vilber Lourmat, Marne la Vallee, France).

### 2.8. Band Stab PCR and Sanger Sequencing

Target bands were excised under UV light using a 200 µL pipette tip and transferred into a PCR master mix containing AmpliTaq Gold DNA Polymerase (Applied Biosystems, Melbourne, Australia) [24]. PCR was performed with a 5 °C lower annealing temperature and an additional 5 cycles compared to the optimised conditions to isolate specific amplicons. The resulting PCR products were purified using Diffinity Rapid Tips (Diffinity Genomics, West Chester, PA, USA) and combined with the corresponding primer. Purified products were sent to the Australian Genome Research Facility (AGRF, Perth, Australia) for Sanger sequencing.

### 2.9. Data and Code Availability

The Sanger sequencing data have been submitted to NCBI and are available under the GenBank accession numbers OR575746, OR575747, and PQ732990. Code used to perform the novel isoform detection and data to generate the figures are available from https://github.com/anuradhareddi/APOC3, accessed on 13 March 2025.

## 3. Results

### 3.1. Long-Read Transcriptome Annotation Reveals Novel APOC3 Isoforms

Given that *APOC3* is predominantly expressed in the liver, we analysed publicly available long-read transcriptomic datasets from immortalised human hepatoma cell lines, HepG2 and Huh7, as well as from healthy liver tissue (Supplementary Table S1) to explore isoform diversity. The FLAMES pipeline was employed for detecting alternative splicing isoforms, while SQANTI3 was used for quality control, classification, and functional annotation of the transcripts.

**Figure 1:**
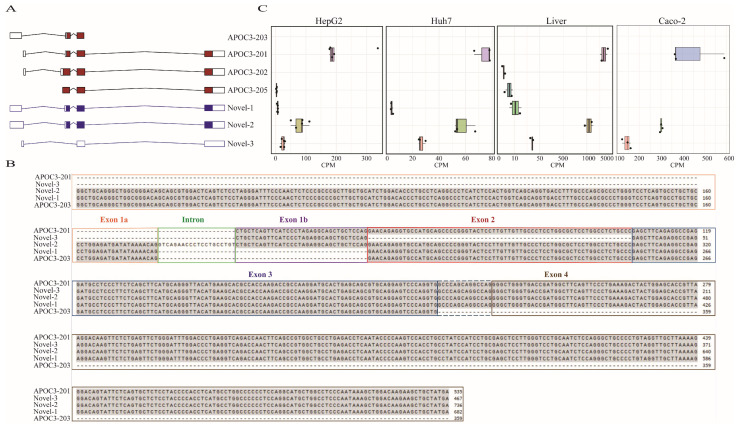
Long-read RNA-seq analysis identifies novel splice isoforms of the *APOC3* gene. (**A**) Schematic representation of *APOC3* splice isoforms, including known isoforms (red) and novel isoforms (blue). Untranslated regions (UTRs) are depicted as open boxes, while coding regions are shown as filled boxes. (**B**) Sequence alignment of the novel isoforms detected through long-read RNA-seq with the *APOC3-203* and *APOC3-201* isoforms. Exon and intron regions are highlighted with coloured rectangular boxes to illustrate structural differences. The dashed box represents the canonical splicing of exon 3. (**C**) Boxplots representing the expression levels of the detected isoforms across HepG2, Huh7, liver tissue, and Caco-2 samples. The y-axis displays the isoform names, which correspond to those shown in panel A and the x-axis represents counts per million (CPM) values.

Our analysis identified three novel isoforms in both cell lines and liver tissue (Figure 1A). All three isoforms (Novel-1, Novel-2, and Novel-3) are splicing variants of the MANE *APOC3-201* transcript. Novel-1 and Novel-2 share exons 2–4 with *APOC3-201*; however, Novel-1 shares exon 1a with *APOC3-203* (Figure 1B), while Novel-2 exhibits an extension of exon 1a into the adjacent intronic region, which merges with exon 1b to form a single exon (Figure 1B). The third isoform, Novel-3, differs from *APOC3-201* by skipping exon 2, which contains the canonical translation start site (Figure 1B). Additionally, we observed a transcript classified as novel in HepG2 that was not detected in Huh7. Several unique transcripts were also identified in liver tissue samples (Supplementary Figure S2); however, they were excluded from further analysis due to lack of reproducibility across datasets.

To explore the expression profiles of these isoforms, we evaluated their relative abundance in both hepatoma cell lines and liver tissue. Among the novel isoforms, Novel-2 was the most abundant, ranking second only to the MANE transcript, while Novel-1 and Novel-3 exhibited consistently lower expression levels across all sample types (Figure 1C). To further classify and functionally annotate these novel isoforms relative to existing annotations, we compared them to reference transcripts. Novel-1 was classified as “novel-in-catalog” and identified as a coding transcript, exhibiting a combination of known splice sites (Table 1). Novel-2 was classified as a “full splice match” with an alternative 5′ end, corresponding to the reference transcript ENST00000227667.8 (*APOC3-201*), and was also identified as a coding transcript with a combination of known junctions (Table 1). Novel 3, also classified as “novel-in-catalog”, was found to be a non-coding transcript with a combination of known splice sites (Table 1).

### 3.2. Extended 5′UTR in Novel-2 Is Associated with More Secondary Structures

**Figure 2:**
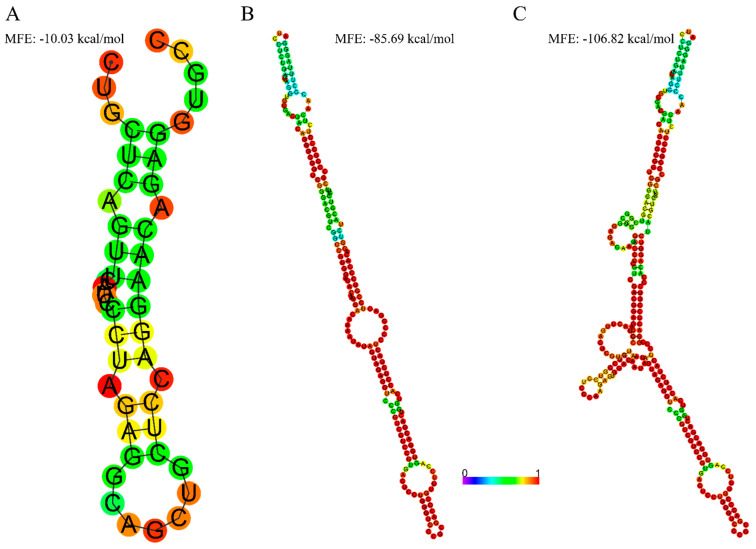
In silico prediction of secondary structures for 5′UTR sequences. The secondary structures were generated using the Vienna RNAfold web server, with minimum free energy (MFE) values indicated for each structure. (**A**) The *APOC3-201* structure forms a relatively simple hairpin loop with an MFE of −10.03 kcal/mol. (**B**) The *APOC3-203* structure adopts a more complex configuration with multiple interior loops and an MFE of −85.69 kcal/mol. (**C**) The Novel-2 structure displays the most intricate configuration with multiple stem-loops and an MFE of −106.82 kcal/mol. The colour bar below represents the base-pairing probability.

Since Novel-2 has a longer 5′UTR, we investigated whether this region forms additional secondary structures using in silico prediction via the RNAfold web server. For comparison, we included the canonical 5′UTR of *APOC3-201* and the alternative 5′UTR of *APOC3-203*. The 5′ UTR of *APOC3-201* exhibited moderate stability with an MFE of −10.03 kcal/mol, indicating a simpler folding pattern (Figure 2A). In contrast, the 5′UTR sequence of *APOC3-203* formed a more complex secondary structure with a lower MFE of −85.69 kcal/mol (Figure 2B). However, the 5′ UTR of Novel-2, which includes the 5′UTR of both *APOC3-203* and *APOC3-201* and the intronic region in between (Figure 1B) displayed the most intricate structure with a significantly lower MFE of −106.82 kcal/mol, suggesting much higher stability of secondary structures (Figure 2C).

### 3.3. Novel Isoforms Are Expressed in Caco-2 Cells

To determine whether the novel *APOC3* isoforms identified in liver-derived samples are expressed in other tissues, we extended our analysis to the small intestine, where *APOC3* is also expressed, albeit to a lesser extent. For this purpose, we analysed long-read sequencing data from intestinal epithelial Caco-2 cells. This analysis revealed the presence of Novel-2 and Novel-3 isoforms, while Novel-1 was not detected under the applied stringent cutoff of 10 counts (Supplementary Figure S2). The expression profiles of Novel-2 and Novel-3 were consistent with hepatic models, with Novel-2 ranking second in abundance after the MANE transcript and Novel-3 ranking third (Figure 1C).

### 3.4. RT-PCR and Sanger Sequencing Confirm Novel APOC3 Isoforms

**Figure 3:**
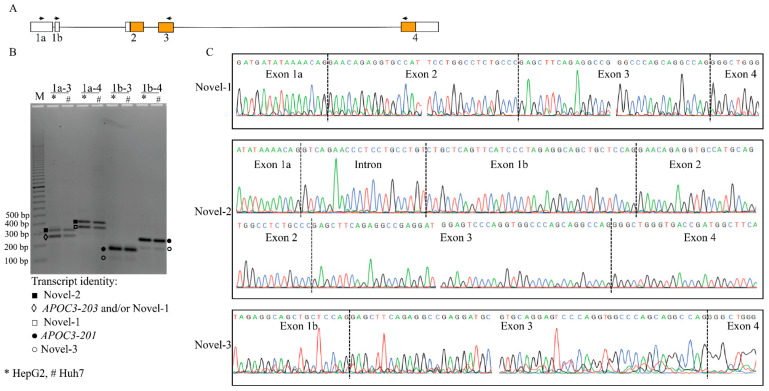
Validation of *APOC3* transcript isoforms using RT-PCR amplification and Sanger sequencing. (**A**) Schematic representation showing the locations of primers (arrows) designed within the exons to detect novel *APOC3* isoforms. (**B**) Gel electrophoresis of amplified *APOC3* transcripts from HepG2 (*) and Huh7 (#) cell lines, with distinct bands corresponding to different transcript isoforms. (**C**) Sanger sequencing chromatograms of the identified *APOC3* transcript isoforms. Black dashed vertical lines indicate exon–exon or exon–intron junctions. Sequencing was performed using both forward and reverse primers to fully resolve splicing junctions. Miscall errors in trace files were manually corrected where necessary. (M—100 bp marker; 1a-3 (exon 1a–exon 3); 1a-4 (exon 1a–exon 4); 1b-3 (exon 1b–exon 3); 1b-4 (exon 1b–exon 4).

To validate the expression of the novel isoforms identified through long-read transcriptomics in cells, we performed RT-PCR using exon-specific primers designed for Novel-1 to -3 (Supplementary Table S1; Figure 3A). Amplification products of the expected sizes were observed in both HepG2 and Huh7 cell lines, confirming the presence of the novel exons (Figure 3B).

Sanger sequencing of the RT-PCR products further validated the inclusion of the novel exons and confirmed the splice junctions predicted by the long-read data (Figure 3C). These results provide strong evidence for the existence of Novel-1 to -3 in hepatoma cell lines. Due to the lack of liver tissue samples, the validation process was limited to hepatoma cell lines.

### 3.5. Novel Isoforms Cluster Closely with Known Human APOC3 Transcripts

To assess the evolutionary conservation of the novel *APOC3* isoforms, we performed a phylogenetic analysis based on sequence similarity (Supplementary Figure S3). The three novel isoforms (Novel-1, Novel-2, and Novel-3) cluster within the human clade, closely with previously annotated human *APOC3* transcripts. Novel-1 is most similar to ENST00000433777.5 (*APOC3-203*), Novel-2 to ENST00000227667.8 (*APOC3-201*; MANE), while Novel-3 forms a separate branch within the human clade, indicating a more distant relationship to known *APOC3* isoforms. None of the novel isoforms group directly with non-human orthologs, suggesting they are unique to humans or have significantly diverged.

## 4. Discussion

In this study, we identified three novel *APOC3* isoforms, expanding the known splicing landscape of this gene. These novel isoforms, Novel-1, Novel-2, and Novel-3, exhibit distinct splicing patterns that may contribute to the regulation of *APOC3* expression. Given the critical role of *APOC3* in triglyceride metabolism and its emerging connection to cardiovascular diseases [1,2], the discovery of these isoforms presents new opportunities for understanding *APOC3* regulation and exploring potential therapeutic interventions.

Our findings were supported by advancements in long-read sequencing technologies, which enable the resolution of complex transcript structures and the detection of previously overlooked or undetected isoforms [25]. Analysis of publicly available long-read sequencing data from hepatoma cell lines (HepG2 and Huh7) and healthy liver tissue revealed the three novel *APOC3* isoforms. These isoforms were likely missed in earlier studies due to the limitations of short-read sequencing, such as insufficient read length or challenges in assembling fragmented reads [26]. The ability of long-read sequencing technologies to capture full-length transcripts, combined with advanced transcript analysis tools, demonstrates their potential to identify novel transcript variants.

The presence of all three novel *APOC3* isoforms in both cancer cell lines and healthy liver tissue indicates that they are not a result of cancer-specific splicing alterations but rather part of the normal *APOC3* transcript repertoire. Novel-1 and Novel-2 share splice junctions of exons 2–4 with the *APOC3-201* transcript but are distinguished by the usage of a longer exon 1 (Figure 1B). This alternative splicing event located within the 5′UTR does not alter the protein coding sequence but may contribute to transcript diversity and regulation of *APOC3* transcript levels.

Interestingly, Novel-1, which includes a longer exon 1a in the 5′UTR similar to that in *APOC3-203*, was either absent or expressed at lower levels than isoforms with the same coding regions (*APOC3-201* and Novel-2) across all analysed samples. The stronger secondary structures predicted by in silico analysis in the 5′UTR region of Novel-1 (identical to *APOC3-203*; Figure 2B) could contribute to slow transcription or reduce transcript stability. These findings align with evidence that alternative splicing within UTRs influences gene expression in at least 13% of mammalian genes [27,28]. For instance, *SERPINA1*, which encodes α-1-antitrypsin, has multiple splicing isoforms that share the same coding sequence but differ in their 5′UTRs, affecting translation efficiency through upstream regulatory elements and secondary structures [29]. Similarly, *BRCA1* expression in certain breast cancers is downregulated by a switch to a longer 5′UTR, which introduces secondary structures or upstream regulatory elements that modulate expression levels [30].

Novel-2, which contains exon 1a but extends farther downstream with the inclusion of adjacent intronic sequences and exon 1b, retains the same splice sites as the MANE transcript. Despite these structural differences, Novel-2 was the second most highly expressed isoform after the MANE transcript, likely due to the preservation of the essential splice architecture found in the MANE transcript. However, its longer 5′UTR may contribute to reduced expression compared to the MANE transcript, as longer 5′UTRs are often associated with increased secondary structures or additional miRNA binding sites, which can interfere with translation initiation or mRNA stability [31,32,33,34]. The secondary structure predictions indicate that the 5′UTR of Novel-2 exhibits greater structural complexity than those of *APOC3-201* and *APOC3-203* (Figure 2C); however, its expression levels remain high, suggesting that additional regulatory mechanisms may mitigate the structural constraints imposed by its extended 5′UTR.

The third isoform, Novel-3, lacks exon 2, which contains the translation initiation site (TIS), potentially disrupting the production of a functional APOC3 protein (Table 1). This alternative splicing event represents a mechanism of unproductive splicing, where the exclusion of critical exons leads to the generation of non-functional or unstable transcripts [35,36,37,38]. Consequently, the absence of TIS likely leads to early degradation of Novel-3, potentially explaining its lower abundance despite having a shorter exon 1 similar to the MANE transcript. Similar regulatory mechanisms have been observed in other genes, such as *PTBP1*, where exon 11 skipping generates an mRNA targeted for degradation by nonsense-mediated decay in a negative feedback loop [37]. Similarly, RNA-binding protein, RBM10, autoregulates its expression by promoting the skipping of essential exons [38], suggesting that unproductive splicing may serve as a broader regulatory strategy, potentially relevant to *APOC3*. The removal of exon 2 from the MANE transcript may represent a mechanism for regulating *APOC3* levels.

Additionally, while exon 2 skipping removes the canonical TIS, Novel-3 retains an in-frame downstream ATG. However, since exon 2 encodes the signal peptide necessary for *APOC3* secretion (Supplementary Figure S4), the loss of exon 2 eliminates this signal peptide, likely impairing protein translocation to the endoplasmic reticulum [39]. As a result, any translated protein would remain in the cells rather than being secreted. This further supports the idea that Novel-3 undergoes unproductive splicing, as the absence of both the TIS and the signal peptide disrupts normal protein synthesis and function, leading to degradation or mislocalisation.

Previous studies have shown that antisense oligonucleotide (ASO) inhibition of *APOC3* expression leads to reduced triglyceride levels and improved hepatic clearance of TRLs [40,41,42]. By promoting the skipping of exon 2, we could increase the expression of Novel-3. Since Novel-3 is non-coding and unable to produce a protein (Table 1), its increased expression could lower functional *APOC3* levels, providing a potential therapeutic strategy for conditions like hypertriglyceridemia, where reducing *APOC3* expression has been shown to be advantageous [42].

The *APOC3* is known to be expressed primarily in liver and to a lesser extent in the small intestine [43]. While our study mainly focused on hepatoma cell lines and healthy liver tissue, we also investigated the expression of *APOC3* isoforms in small intestine models, specifically Caco-2 cells. Our analysis suggests that these isoforms are present in both the liver and small intestine. However, due to the unavailability of liver or small intestine tissue samples, RT-PCR validation was confined to hepatoma cell lines. Nonetheless, our long-read RNA-seq analysis confirmed the presence of these isoforms and supported their expression in both liver tissue and small intestinal models. Further studies involving additional pathological tissues or disease models could provide additional insights into whether these isoforms are more broadly expressed across various conditions. The new isoforms identified in this study were not previously reported and do not cluster with non-human orthologs, suggesting that these alternative splicing events may be unique to humans or have evolved recently in primates.

Although this study provides valuable insights into the novel *APOC3* isoforms, there are some limitations. First, while RNA-seq identified these isoforms, RT-PCR validation was limited to hepatoma cell lines due to the unavailability of primary tissue samples. Further experiments using primary tissue samples from both healthy and disease contexts are necessary to validate the expression of these isoforms and evaluate their relevance across different conditions. Second, although we identified Novel-3 as a non-coding isoform, additional experimental validation is required to determine its role as a regulatory non-coding transcript or its susceptibility to nonsense-mediated decay. Lastly, while our study discovered novel isoforms with distinct splicing patterns, their functional roles in lipid metabolism and cardiovascular disease need to be experimentally verified, particularly in disease models and therapeutic contexts.

## 5. Conclusions

In conclusion, we identified three novel *APOC3* isoforms with distinct splicing patterns that may influence *APOC3* expression and lipid metabolism. The presence of alternative 5′UTRs and a non-coding isoform suggests potential mechanisms that could regulate transcript stability and translational efficiency. Since *APOC3* is a key regulator of triglyceride metabolism and cardiovascular risk, these findings provide new opportunities for therapeutic strategies, particularly through ASO-mediated modulation of splicing. The discovery of a non-coding isoform (Novel-3) suggests that promoting exon 2 skipping could serve as a strategy to lower functional *APOC3* levels, with potential benefits for hypertriglyceridemia treatment. Additionally, the presence of these isoforms in both hepatic and intestinal models suggests broader physiological relevance. While further research is required to fully understand their functional roles, these novel isoforms offer the potential to explore therapeutic strategies.

## Figures and Tables

**Table 1:** Classification and functional annotation of novel *APOC3* isoforms using SQANTI3.

Isoform	Structural_Category	Associated_Transcript	Coding	Subcategory
Novel-1	novel_in_catalog	novel	coding	combination_of_known_junctions
Novel-2	full-splice_match	ENST00000227667.8	coding	alternative_5end
Novel-3	novel_in_catalog	novel	non_coding	combination_of_known_splicesites

## Data Availability

The Sanger sequencing data has been submitted to NCBI and is available under the GenBank accession numbers OR575746, OR575747, and PQ732990. Code used to perform the novel isoform detection and data to generate the figures is available from https://github.com/anuradhareddi/APOC3, accessed on 13 March 2025.

## Supplementary Materials

The following supporting information can be downloaded at: https://www.mdpi.com/article/10.3390/genes16040412/s1, Figure S1. Schematic representation of the bioinformatics workflow and validation process for identifying and analysing novel *APOC3* isoforms. Figure S2. Transcript structure of *APOC3* isoforms identified in hepatoma cell lines, liver tissue, and Caco-2 cells. Figure S3. Comparative analysis of human *APOC3* isoforms and their orthologs. Figure S4. Open reading frames (ORFs) of the MANE transcript and Novel-3 isoform of *APOC3*. Table S1. RNA sequencing dataset used for analysis. Table S2. RT-PCR primers used for validation of novel *APOC3* isoforms.

## References

[1] Sarwar N., Danesh J., Eiriksdottir G., Sigurdsson G., Wareham N., Bingham S., Boekholdt S.M., Khaw K.-T., Gudnason V. (2007). Triglycerides and the risk of coronary heart disease: 10,158 incident cases among 262,525 participants in 29 Western prospective studies. Circulation.

[2] Austin M.A., Hokanson J.E., Edwards K.L. (1998). Hypertriglyceridemia as a cardiovascular risk factor. Am. J. Cardiol..

[3] Kersten S. (2014). Physiological regulation of lipoprotein lipase. Biochim. Biophys. Acta.

[4] Ding Y., Wang Y., Zhu H., Fan J., Yu L., Liu G., Liu E. (2011). Hypertriglyceridemia and delayed clearance of fat load in transgenic rabbits expressing human apolipoprotein CIII. Transgenic Res..

[5] Jørgensen A.B., Frikke-Schmidt R., Nordestgaard B.G., Tybjærg-Hansen A. (2014). Loss-of-function mutations in APOC3 and risk of ischemic vascular disease. N. Engl. J. Med..

[6] Crosby J., Peloso G.M., Auer P.L., Crosslin D.R., Stitziel N.O., Lange L.A., Lu Y., Tang Z.Z., Zhang H., Hindy G. (2014). Loss-of-function mutations in APOC3, triglycerides, and coronary disease. N. Engl. J. Med..

[7] Pollin T.I., Damcott C.M., Shen H., Ott S.H., Shelton J., Horenstein R.B., Post W., McLenithan J.C., Bielak L.F., Peyser P.A. (2008). A null mutation in human APOC3 confers a favorable plasma lipid profile and apparent cardioprotection. Science.

[8] Ito Y., Azrolan N., O’Connell A., Walsh A., Breslow J.L. (1990). Hypertriglyceridemia as a Result of Human apo CII Gene Expression in Transgenic Mice. Science.

[9] Maeda N., Li H., Lee D., Oliver P., Quarfordt S., Osada J. (1994). Targeted disruption of the apolipoprotein C-III gene in mice results in hypotriglyceridemia and protection from postprandial hypertriglyceridemia. J. Biol. Chem..

[10] Qamar A., Khetarpal S.A., Khera A.V., Qasim A., Rader D.J., Reilly M.P. (2015). Plasma Apolipoprotein C-III Levels, Triglycerides, and Coronary Artery Calcification in Type 2 Diabetics. Arterioscler. Thromb. Vasc. Biol..

[11] Åvall K., Ali Y., Leibiger I.B., Leibiger B., Moede T., Paschen M., Dicker A., Daré E., Köhler M., Ilegems E. (2015). Apolipoprotein CIII links islet insulin resistance to β-cell failure in diabetes. Proc. Natl. Acad. Sci. USA.

[12] Bozzetto L., Berntzen B.J., Kaprio J., Rissanen A., Taskinen M.-R., Pietiläinen K.H. (2020). A higher glycemic response to oral glucose is associated with higher plasma apolipoprotein C3 independently of BMI in healthy twins. Nutr. Metab. Cardiovasc. Dis..

[13] Sol E.-r.M., Sundsten T., Bergsten P. (2009). Role of MAPK in apolipoprotein CIII-induced apoptosis in INS-1E cells. Lipids Health Dis..

[14] Kawakami A., Aikawa M., Alcaide P., Luscinskas F.W., Libby P., Sacks F.M. (2006). Apolipoprotein CIII Induces Expression of Vascular Cell Adhesion Molecule-1 in Vascular Endothelial Cells and Increases Adhesion of Monocytic Cells. Circulation.

[15] Harrison P.W., Amode M.R., Austine-Orimoloye O., Azov A.G., Barba M., Barnes I., Becker A., Bennett R., Berry A., Bhai J. (2023). Ensembl 2024. Nucleic Acids Res..

[16] Wu J., Hu W., Li S. (2023). Long-read transcriptome sequencing reveals allele-specific variants at high resolution. Trends Genet..

[17] Glinos D.A., Garborcauskas G., Hoffman P., Ehsan N., Jiang L., Gokden A., Dai X., Aguet F., Brown K.L., Garimella K. (2022). Transcriptome variation in human tissues revealed by long-read sequencing. Nature.

[18] Pyatnitskiy M.A., Arzumanian V.A., Radko S.P., Ptitsyn K.G., Vakhrushev I.V., Poverennaya E.V., Ponomarenko E.A. (2021). Oxford Nanopore MinION Direct RNA-Seq for Systems Biology. Biology.

[19] Sarygina E., Kozlova A., Deinichenko K., Radko S., Ptitsyn K., Khmeleva S., Kurbatov L.K., Spirin P., Prassolov V.S., Ilgisonis E. (2023). Principal Component Analysis of Alternative Splicing Profiles Revealed by Long-Read ONT Sequencing in Human Liver Tissue and Hepatocyte-Derived HepG2 and Huh7 Cell Lines. Int. J. Mol. Sci..

[20] Kaur G., Perteghella T., Carbonell-Sala S., Gonzalez-Martinez J., Hunt T., Mądry T., Jungreis I., Arnan C., Lagarde J., Borsari B. (2024). GENCODE: Massively expanding the lncRNA catalog through capture long-read RNA sequencing. bioRxiv.

[21] Yang J., Hirai Y., Iida K., Ito S., Trumm M., Terada S., Sakai R., Tsuchiya T., Tabata O., Kamei K.-I. (2023). Integrated-gut-liver-on-a-chip platform as an in vitro human model of non-alcoholic fatty liver disease. Commun. Biol..

[22] Pardo-Palacios F.J., Arzalluz-Luque A., Kondratova L., Salguero P., Mestre-Tomás J., Amorín R., Estevan-Morió E., Liu T., Nanni A., McIntyre L. (2024). SQANTI3: Curation of long-read transcriptomes for accurate identification of known and novel isoforms. Nat. Methods.

[23] Gruber A.R., Lorenz R., Bernhart S.H., Neuböck R., Hofacker I.L. (2008). The Vienna RNA websuite. Nucleic Acids Res..

[24] Wilton S.D., Lim L., Dye D., Laing N. (1997). Bandstab: A PCR-based alternative to cloning PCR products. Biotechniques.

[25] Ebert P., Audano P.A., Zhu Q., Rodriguez-Martin B., Porubsky D., Bonder M.J., Sulovari A., Ebler J., Zhou W., Mari R.S. (2021). Haplotype-resolved diverse human genomes and integrated analysis of structural variation. Science.

[26] Steijger T., Abril J.F., Engström P.G., Kokocinski F., Hubbard T.J., Guigó R., Harrow J., Bertone P., The RGASP Consortium (2013). Assessment of transcript reconstruction methods for RNA-seq. Nat. Methods.

[27] Carninci P., Kasukawa T., Katayama S., Gough J., Frith M.C., Maeda N., Oyama R., Ravasi T., Lenhard B., Wells C. (2005). The Transcriptional Landscape of the Mammalian Genome. Science.

[28] Eden E., Brunak S. (2004). Analysis and recognition of 5′ UTR intron splice sites in human pre-mRNA. Nucleic Acids Res..

[29] Corley M., Solem A., Phillips G., Lackey L., Ziehr B., Vincent H.A., Mustoe A.M., Ramos S.B.V., Weeks K.M., Moorman N.J. (2017). An RNA structure-mediated, posttranscriptional model of human α-1-antitrypsin expression. Proc. Natl. Acad. Sci. USA.

[30] Sobczak K., Krzyzosiak W.J. (2002). Structural Determinants of BRCA1 Translational Regulation. J. Biol. Chem..

[31] Pelletier J., Sonenberg N. (1985). Insertion mutagenesis to increase secondary structure within the 5′ noncoding region of a eukaryotic mRNA reduces translational efficiency. Cell.

[32] Zhan Y., Hu Z., Lu Z., Lin Z. (2024). The evolution of 5′ UTR length was shaped by natural selection for its functional impact on gene expression. bioRxiv.

[33] Wieder N., D’souza E.N., Martin-Geary A.C., Lassen F.H., Talbot-Martin J., Fernandes M., Chothani S.P., Rackham O.J.L., Schafer S., Aspden J.L. (2024). Differences in 5′untranslated regions highlight the importance of translational regulation of dosage sensitive genes. Genome Biol..

[34] Nitschke L., Tewari A., Coffin S.L., Xhako E., Pang K., Gennarino V.A., Johnson J.L., Blanco F.A., Liu Z., Zoghbi H.Y. (2020). miR760 regulates ATXN1 levels via interaction with its 5′ untranslated region. Genes Dev..

[35] Sureau A., Gattoni R., Dooghe Y., Stévenin J., Soret J. (2001). SC35 autoregulates its expression by promoting splicing events that destabilize its mRNAs. EMBO J..

[36] Zhou Y., Liu S., Liu G., Oztürk A., Hicks G.G. (2013). ALS-associated FUS mutations result in compromised FUS alternative splicing and autoregulation. PLoS Genet..

[37] Wollerton M.C., Gooding C., Wagner E.J., Garcia-Blanco M.A., Smith C.W. (2004). Autoregulation of polypyrimidine tract binding protein by alternative splicing leading to nonsense-mediated decay. Mol. Cell.

[38] Sun Y., Bao Y., Han W., Song F., Shen X., Zhao J., Zuo J., Saffen D., Chen W., Wang Z. (2017). Autoregulation of RBM10 and cross-regulation of RBM10/RBM5 via alternative splicing-coupled nonsense-mediated decay. Nucleic Acids Res..

[39] Jong M.C., Hofker M.H., Havekes L.M. (1999). Role of ApoCs in Lipoprotein Metabolism. Arterioscler. Thromb. Vasc. Biol..

[40] Graham M.J., Lee R.G., Bell T.A., Fu W., Mullick A.E., Alexander V.J., Singleton W., Viney N., Geary R., Su J. (2013). Antisense oligonucleotide inhibition of apolipoprotein C-III reduces plasma triglycerides in rodents, nonhuman primates, and humans. Circ. Res..

[41] Gordts P.L., Nock R., Son N.-H., Ramms B., Lew I., Gonzales J.C., Thacker B.E., Basu D., Lee R.G., Mullick A.E. (2016). ApoC-III inhibits clearance of triglyceride-rich lipoproteins through LDL family receptors. J. Clin. Investig..

[42] Ramms B., Patel S., Sun X., Pessentheiner A.R., Ducasa G.M., Mullick A.E., Lee R.G., Crooke R.M., Tsimikas S., Witztum J.L. (2022). Interventional hepatic apoC-III knockdown improves atherosclerotic plaque stability and remodeling by triglyceride lowering. JCI Insight.

[43] Wu A.L., Windmueller H.G. (1978). Identification of circulating apolipoproteins synthesized by rat small intestine in vivo. J. Biol. Chem..

