# Supplementary material for: Discovery of Novel APOC3 Isoforms in Hepatic and Intestinal Cell Models Using Long-Read RNA Sequencing
Source: Genes (Basel). 2025 Mar 31;16(4):412. doi: 10.3390/genes16040412 (PMC12027394; doi:10.3390/genes16040412)
Supplement: Supplementary file 1 [file genes-16-00412-s001.zip › genes-3556488-supplementary.pdf]

## Supplementary Data

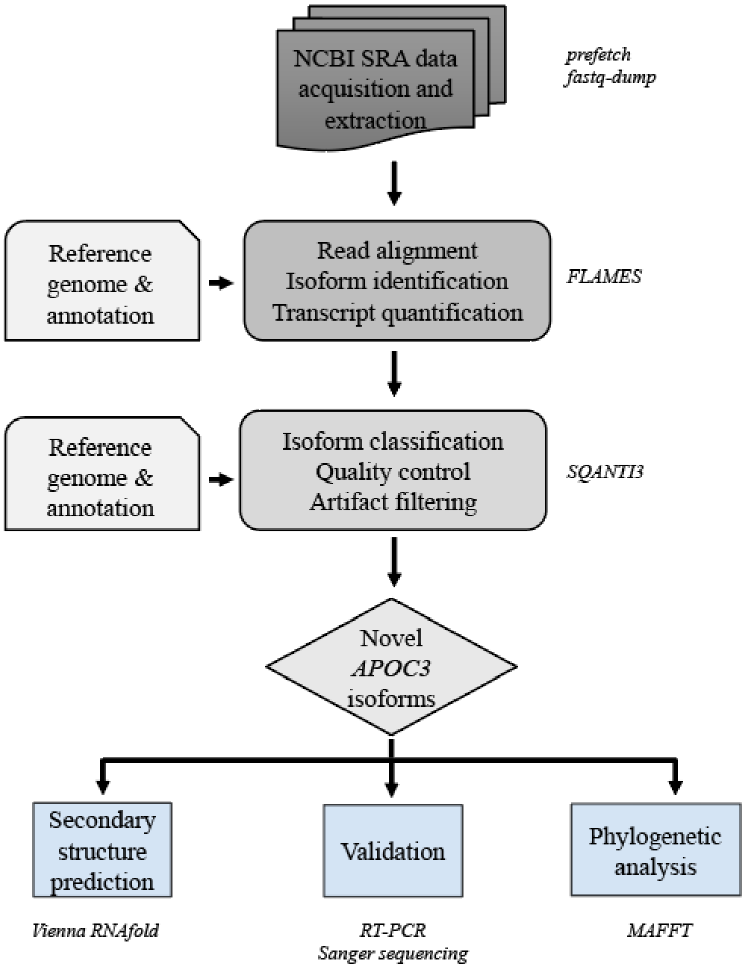


**Supplementary Figure S1.** Schematic representation of the bioinformatics workflow and validation process for identifying and analysing novel *APOC3* isoforms.

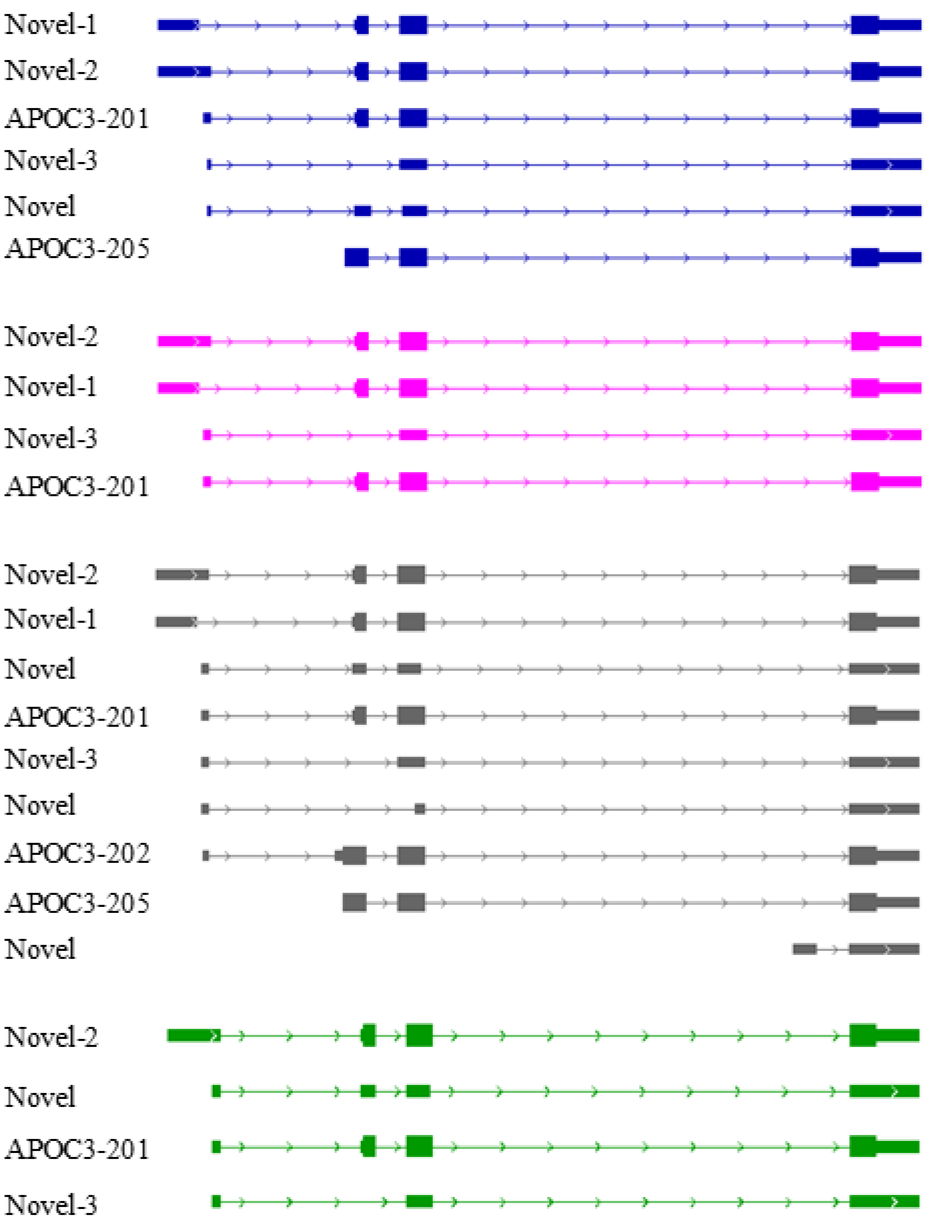


**Supplementary Figure S2.** Transcript structure of *APOC3* isoforms identified in hepatoma cell lines, liver tissue, and Caco-2 cells. Variations in splicing patterns are shown across different sample types, with isoforms identified in HepG2 (blue), Huh7 (pink), liver tissue (grey), and Caco-2 cells (green). Novel isoforms are displayed alongside the known reference transcripts expressed in each sample. Exons are represented as boxes, with coding exons shown in thicker boxes and UTR in thinner boxes. Arrows indicate the direction of transcription.

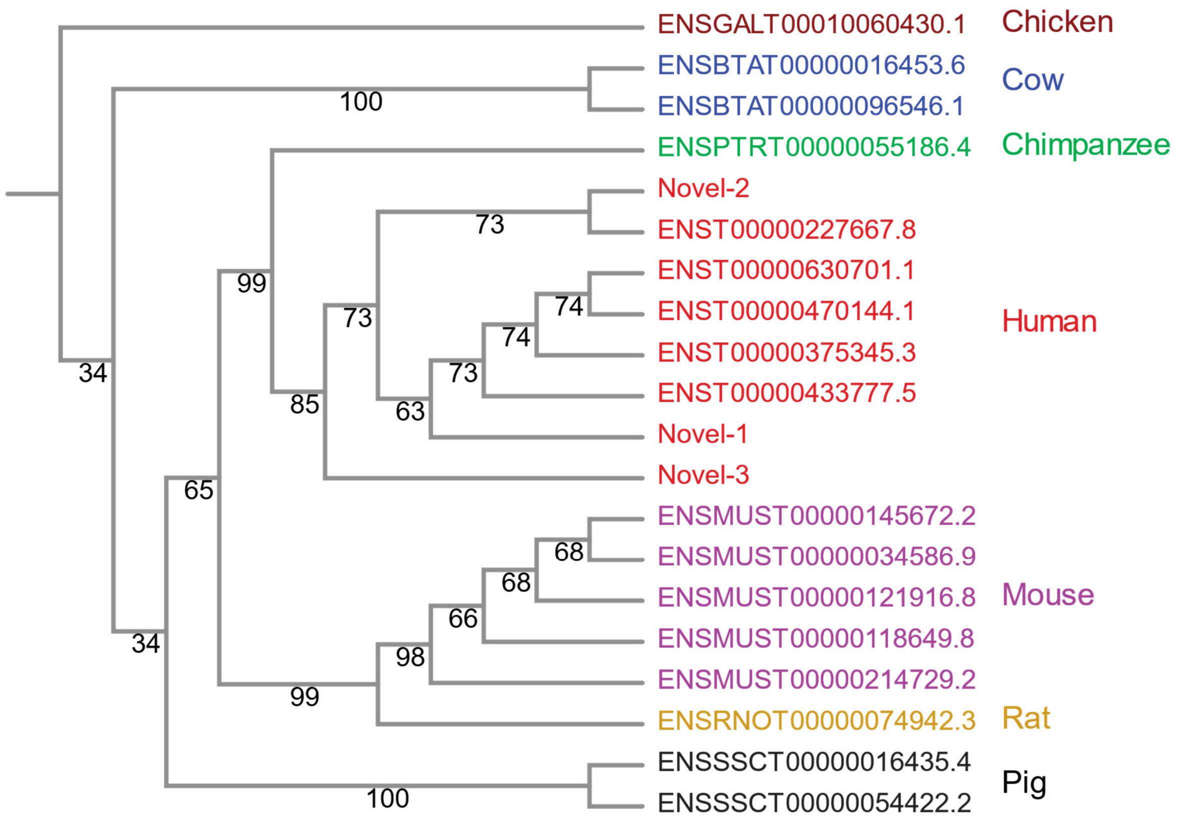


**Supplementary Figure S3.** Comparative analysis of human *APOC3* isoforms and their orthologs. The phylogenetic tree illustrates the evolutionary relationships of *APOC3* isoforms from various species, including human (red), chimpanzee (green), cow (blue), chicken (brown), mouse (purple), rat (yellow), and pig (black). Novel human isoforms (Novel-1, Novel-2, and Novel-3) are incorporated to assess their evolutionary conservation. The clustering is based on sequence similarity, with closely related transcripts grouped together. The values on branches represent bootstrap values.

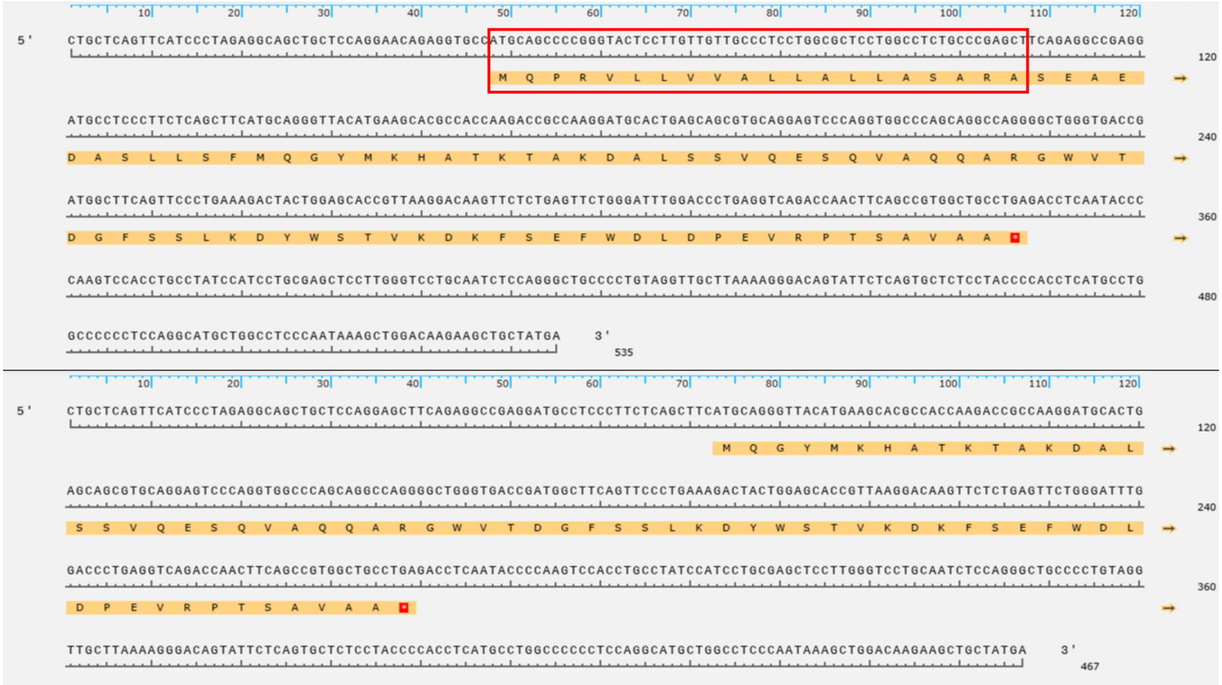

**Supplementary Figure S4.** Open reading frames (ORFs) of the MANE transcript and Novel-3 isoform of *APOC3*. The top panel displays the MANE transcript, with the signal peptide region highlighted in a red rectangle. The bottom panel shows the Novel-3 isoform, which results from exon 2 skipping, remaining in-frame with an alternative ORF but losing the signal peptide. The translated protein sequences are highlighted in yellow, and stop codons are indicated by asterisks (*).

**Supplementary Table S1.** RNA sequencing dataset used for analysis.

| *SRA#* | *Sample* | *Instrument* | *Selection* | *Reference* |
|---|---|---|---|---|
| SRR16071311 | HepG2 | OXFORD_NANOPORE (MinION) | direct RNA | [18] |
| SRR16071315 | HepG2 | OXFORD_NANOPORE (MinION) | direct RNA | [18] |
| SRR16071316 | HepG2 | OXFORD_NANOPORE (MinION) | direct RNA | [18] |
| SRR16071317 | HepG2 | OXFORD_NANOPORE (MinION) | direct RNA | [18] |
| SRR16071318 | HepG2 | OXFORD_NANOPORE (MinION) | direct RNA | [18] |
| SRR22020440 | Huh7 | OXFORD_NANOPORE (MinION) | direct RNA | [19] |
| SRR22020441 | Huh7 | OXFORD_NANOPORE (MinION) | direct RNA | [19] |
| SRR22020442 | Huh7 | OXFORD_NANOPORE (MinION) | direct RNA | [19] |
| ERR13885915 | Liver | OXFORD_NANOPORE (MinION) | FL-cDNA | [20] |
| ERR13885921 | Liver | OXFORD_NANOPORE (MinION) | FL-cDNA | [20] |
| SRR19744313 | Caco-2 | OXFORD_NANOPORE (MinION) | cDNA | [21] |
| SRR19744314 | Caco-2 | OXFORD_NANOPORE (MinION) | cDNA | [21] |
| SRR19744315 | Caco-2 | OXFORD_NANOPORE (MinION) | cDNA | [21] |

**Supplementary Table S2.** RT-PCR primers used for validation of novel *APOC3* isoforms.

| **Primer Name** | **Sequence (5’ – 3’)** |
|---|---|
| hAPOC3_Ex1.bF | TTC ATC CCT AGA GGC AGC TG |
| hAPOC3_Ex1.aF | TTG CTG CAT CTG GAC ACC CT |
| hAPOC3_Ex3R | ACG CTG CTC AGT GCA TCC TT |

| | |
|---|---|
| hAPOC3_Ex4R | GTC CTT AAC GGT GCT CCA GT |
